# Delusion of pregnancy: Clinical case

**DOI:** 10.1192/j.eurpsy.2021.2102

**Published:** 2021-08-13

**Authors:** M.F. Tascon Guerra, B. Díaz Marqués, G. Manrique Ovejero

**Affiliations:** 1 Psychiatry, Hospital Ntra Sra del Prado, Talavera de la Reina, Toledo, Spain; 2 Family Medicine, Hospital Ntra Sra del Prado, Talavera de la Reina, Toledo, Spain

**Keywords:** pregnancy, Pseudocyesis, Pseudopregnancy, Delusion

## Abstract

**Introduction:**

Delusion of pregnancy has been described as a false and fixed belief of being pregnant despite factual evidence to the contrary. Pseudocyesis is a condition in which the patient has all signs and symptoms of pregnancy except for the confirmation of the presence of a fetus. There has been described symptoms as abdominal distention, cessation of menses, morning sickness, etc.

**Objectives:**

The aim of this work was to distinguish between pseudocyesis and pseudopregnancy. This case presents a single 49-year-old woman, who developed the delusion of being pregnant after months of lonely confinement during Covid-19 quarantine.

**Methods:**

She had missed her period for 10 months. She was convinced that she could feel the fetal movements. Her thought content revealed delusion of persecution, reference, and delusion of being pregnant. She did not reveal any hallucinations. Blood tests and brain imaging revealed no abnormalities. □The treatment was started with Paliperidone 100mg/month, and clonazepam 2mg/d.

**Results:**

The patient showed a substantial improvement within 10 weeks of treatment. Pseudocyesis and delusional pregnancy have been rarely described on scientific bibliography. A distinction has been demonstrated based on the consideration of the associated psychotic features that might be present in delusions of pregnancy, which were described in the current case. On the other hand, pseudocyesis clinical presentation is centered on the false signs and symptoms of pregnancy.

**Conclusions:**

Antipsychotics played a key role in the delusion of pregnancy. Psychodynamic and supportive psychotherapy could play a pivotal role in the management of pseudocyesis.

**Disclosure:**

No significant relationships.

